# Microwave-Assisted Oxalic Acid Pretreatment for the Enhancing of Enzyme Hydrolysis in the Production of Xylose and Arabinose from Bagasse

**DOI:** 10.3390/molecules23040862

**Published:** 2018-04-10

**Authors:** Yuhuan Yan, Chunhui Zhang, Qixuan Lin, Xiaohui Wang, Banggui Cheng, Huiling Li, Junli Ren

**Affiliations:** 1 School of Light Industry and Engineering, South China University of Technology, Guangzhou 510640, China; paperscience@yanyuhuan.com (Y.Y.); linqixuan7@163.com (Q.L.); chengbg2669871474@163.com (B.C.); 2 State Key Laboratory of Pulp and Paper Engineering, South China University of Technology, Guangzhou 510640, China; wxhui006@163.com; 3 Guangdong Key Laboratory for Innovative Development and Utilization of Forest Plant Germplasm, State Key Laboratory for Conservation and Utilization of Subtropical Agro-bioresources, South China Agricultural University, Guangzhou 510642, China; lihl@scau.edu.cn

**Keywords:** bagasse, microwave-assisted acid pretreatment, oxalic acid, xylose, arabinose, enzymatic hydrolysis

## Abstract

In this study, highly-efficient hydrolysis of bagasse into xylose and arabinose sugars (C5 sugars) was developed by microwave-assisted oxalic acid pretreatment under mild reaction conditions. The effects of acid and hydrolysis conditions on the C5 sugar yields were discussed. The results showed that oxalic acid performed better than hydrochloric acid and maleic acid, and was a promising alternative to sulfuric acid for xylose production at the same acid concentration. The maximum yields of xylose (95.7%) and arabinose (91.5%) were achieved via the microwave-assisted oxalic acid pretreatment (120 °C, 10 min, 0.4 mol/L, solid–liquid ratio of 1:50 g/mL), indicating that almost all xylan-type hemicelluloses were released from the cell wall and hydrolyzed into C5 sugars. After pretreatment, more than 90% of the cellulose in the residual bagasse was converted to glucose (92.2%) by enzymatic hydrolysis. This approach could realize the highly-efficient hydrolysis of xylan from bagasse into C5 sugars, which would enhance the enzyme hydrolysis of treated bagasse into glucose.

## 1. Introduction

Lignocellulosic biomass, including herbaceous plants, wood, agricultural crops, and their residues after processing, is regarded as a promising alternative renewable resource for the replacement of traditional fossil resources [1]. Among these different types of biomass, bagasse has attracted much interest because it is considered as one of the significant second-generation biomass materials. As the main byproduct of the sugar industry, bagasse is mainly composed of cellulose, hemicellulose, and lignin. China is the third-largest country in terms of sugarcane cultivation, after Brazil and India, in which the total annual output of sugarcane is more than 123,000,000 tons [2], and the production capacity of dry bagasse is approximately 16,770,000 tons. Not only does Bagasse contain biomass energy, it is also a good source of natural polymeric materials and green chemicals. More than 50% of bagasse is recycled to generate power and heat, whereas the rest can be used as culture media for edible fungi, as a raw material for functional food production, or for use in the biorefinery industry.

Bagasse can be converted into commercial sugars, for instance, d-xylose, d-glucose, and l-arabinose [3,4,5], which could be further converted into biofuels and important platform chemicals, such as furfural and hydroxymethyl furfural (HMF). However, efficient production of these sugars is of great importance to their utilization. Bagasse has a complex and rigid structure, in which cellulose constitutes the framework of the cell wall in the form of protofibril, while lignin and hemicellulose are wrapped around protofibril to protect cellulose [4]. To overcome recalcitrance, the ultrastructure of the plant cell wall needs to be broken down, and the lignin shield needs to be opened up to access the carbohydrates. Several physical, chemical, and microbiological pretreatment techniques have been developed to destroy the lignocellulose structure [6,7] and increase the accessible surface area of the cellulose for further conversion [8].

Inorganic acids (sulfuric acid, hydrochloric acid, phosphoric acid, etc.) are commonly used to hydrolyze lignocellulosic biomass [9,10]. However, there are some inherent disadvantages, such as equipment corrosion and low reaction selectivity [11]. Dilute sulfuric acid pretreatment (50–300 mmol/L) at 100–200 °C has usually been applied to disrupt the lignin–carbohydrate matrix [12,13,14]. However, the generation of inhibitors during this process is unfavorable for subsequent enzymatic hydrolysis and yeast fermentation [15]. To overcome these limitations, more attention has been drawn towards organic acids that could be applied to selectively depolymerize hemicelluloses [16]. Maleic and oxalic acids can also effectively disrupt the lignocellulosic structure. High sugar yields (>90%, xylose and glucose) were achieved when wheat straw was processed by organic acid pretreatment (at >190 °C) and subsequent enzymatic hydrolysis. The highest total xylose yield (monomer and oligomer), of approximately 84%, was obtained when maple wood was pretreated with 0.5% oxalic or sulfuric acid at 160 °C for approximately 30 min [17]. It has been proven that oxalic acid pretreatment could be a promising alternative to sulfuric acid pretreatment. Previous studies showed that dicarboxylic acids had dramatic chemical and practical characteristics: (1) selective hydrolysis of hemicellulose in lignocellulose [18]; (2) controlled stepwise acidity which facilitates more efficient hydrolysis of the substrate, over a wider range of temperature and pH values [11]; and (3) limited corrosive behavior, easy handling and storage when compared to sulfuric acid. Generally, high temperature and/or long time periods have to be used to get high sugar yields during pretreatment. There is still an urgent problem to be solved regarding the efficient conversion of biomass-derived carbohydrates under mild conditions.

Microwave irradiation is a pivotal and fast developing technology in green chemistry [19], and recently it has been employed to improve reaction rates, shorten reaction time, and lessen the formation of side products [20]. The principle of this approach applies an alternative electromagnetic field to provoke intermolecular friction between polar molecules, thus releasing kinetic energy in the inner media by rapid heating [20]. The energy is directly supplied to the biomass in the form of microwave radiation, which is then converted into heat. This technology has been applied to the study of sugarcane–bagasse conversion [21,22], and was originally proposed for use in the depolymerization of lignocellulose into sugars [23].

In this research, a feasible approach was developed to selectively hydrolyze bagasse into C5 sugars (xylose and arabinose) by microwave-assisted organic acid pretreatment. Different kinds of acids were comparatively studied for depolymerization of xylan in bagasse. The influence of this pretreatment on the enzymatic hydrolysis of treated bagasse was also discussed. The physical and chemical properties of bagasse and the pretreated solid residues were characterized by scanning electron micrography (SEM) and thermal gravity analysis (TGA). The C5 sugars and glucose produced by the two-step treatment of bagasse could be converted into ethanol, butanol, or platform chemicals, such as furfural, HMF, and levulinic acid.

## 2. Results and Discussion

### 2.1. Influence of Different Acids on Xylose Yield

**Figure 1:**
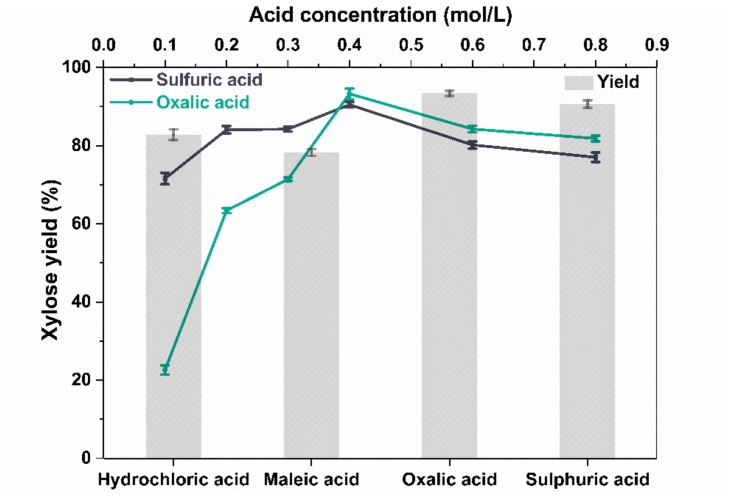
Effect of acid concentrations and types on xylose yield (120 °C, 10 min, a solid–liquid ratio of 1:20; bar chart 0.4 mol/L; line chart 0.1–0.8 mol/L).

Xylose is a pentose, and the main component in xylan-type hemicelluloses. The xylose yields from bagasse, after microwave-assisted acid pretreatments (120 °C, 10 min, solid–liquid ratio 1:20), are shown in Figure 1 (Bar Chart). The experiments were conducted at the same acid concentration (0.4 mol/L) for hydrochloric acid, maleic acid, oxalic acid, and sulfuric acid. The highest xylose yield was obtained during the oxalic acid pretreatment, which indicated that oxalic acid displayed an excellent catalytic performance in the hydrolysis of bagasse into xylose at the given conditions, due to its dicarboxylic property [24] and superior selectivity for hemicellulose hydrolysis to form xylose [25].

The pKa1 values of hydrochloric acid (−8) and sulfuric acid (−3) are much lower than oxalic acid (1.23) [26,27]. However, the xylose yields obtained from the inorganic acid (hydrochloric acid and sulfuric acid) pretreatments of bagasse were slightly lower than that of the oxalic acid pretreatment at a concentration of 0.4 mol/L. This was due to the strong acidity of the inorganic acids, which led to strong side reactions [15,28]. Similar results were observed in the case where maple wood was pretreated with oxalic acid and inorganic acids [17]. In comparison, oxalic acid performed better than maleic acid under our experimental conditions, although they were both dominant di-acid species. According to the pKa1 values of oxalic acid and maleic acid (1.93), the acid strength of oxalic acid was slightly higher than maleic acid, indicating the acidity of the former was more beneficial to fractionation of xylose from bagasse. 

The catalytic performance of sulfuric acid and oxalic acid at different concentrations (0.1–0.8 mol/L) is illustrated in the line chart of Figure 1. The microwave-assisted acid pretreatments were carried out at 120 °C for 10 min with a solid–liquid ratio of 1:20. The xylose yields were similar for the two acids used in pretreatment. The value of 0.4 mol/L seemed to be an optimal acid concentration because the xylose yield reached its highest level for both acids (sulfuric acid, 90.5%; oxalic acid, 93.2%) at this concentration. A higher acid concentration could result in the dehydration of xylose to the undesired furfural [29]. In addition, when the acid concentration was lower than 0.4 mol/L, the xylose yield from the sulfuric acid pretreatment was higher than that using oxalic acid, because the former was the stronger acid and thus could depolymerize xylan into xylose at lower concentrations. In previous research, it has been shown that sulfuric acid has induced higher values in the rate constant for xylose degradation to furfural than oxalic acid [30]. Therefore, sulfuric acid produced sugar degradation products more easily than oxalic acid, especially at higher concentrations. Additionally, oxalic acid could be precipitated from the aqueous phase by adding CaSO_4_ or Ca(OH)_2_, and after acidification oxalic acid could be re-formed.

### 2.2. Influence of Temperature and Time on C5 and C6 Sugars Yield

**Figure 2:**
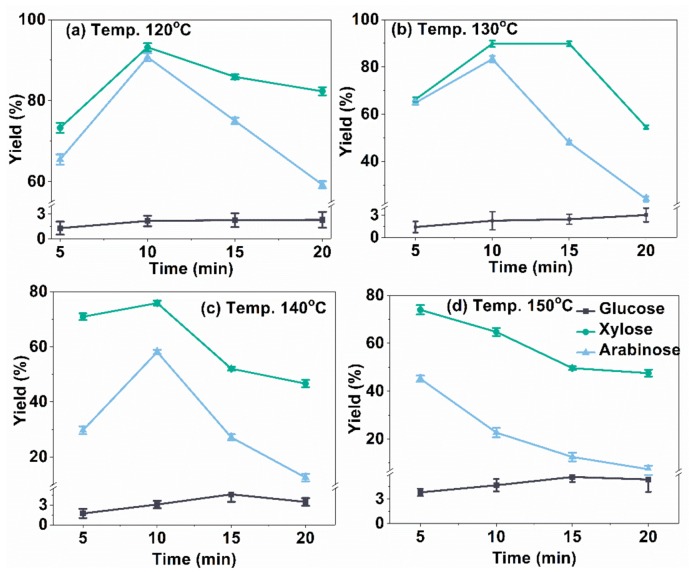
The effect of the reaction temperature and time on sugar yields (oxalic acid, 0.4 mol/L, solid–liquid ratio 1:20).

The effects of reaction temperature (120, 130, 140, and 150 °C) and time (5, 10, 15, and 20 min) on the yield of C5 and C6 sugars during microwave-assisted acid pretreatments are illustrated in Figure 2. Experiments were conducted in a closed-vessel microwave reactor (1 g of dried bagasse, 20 mL of 0.4 mol/L oxalic acid). At low temperatures (below 150 °C), the xylose yield reached a maximum level within 10 min, while the yield gradually decreased with increasing time at 150 °C. This might be ascribed to the side-reactions caused by higher temperature [31]. Another possible reason was that oxalic acid was partially decomposed into H_2_O, CO_2_, and CO at temperature above 150 °C. The highest xylose yield (93.2%) was obtained during the pretreatment at 120 °C for 10 min, implying that almost all of the xylan in the bagasse was released and depolymerized into xylose sugar, under the given pretreatment conditions.

The trend of arabinose yield was similar to that of xylose, and the maximum level of 90.7% was also obtained with treatment at 120 °C for 10 min. It was reported that the hemicellulose of sugarcane bagasse was composed mainly of l-arabino-(4-*O*-methyl-d-glucurono) xylan [32,33]. As l-arabinose was the branched chain of the hemicelluloses, it could easily be broken down, especially when the main chain was destroyed [34,35]. Thus, most of the arabinose could be released from the bagasse within 10 min. However, high temperature could cause further degradation of the released arabinose.

Unlike xylose and arabinose, the trend of glucose yield was rather different. Firstly, the yields of glucose were far lower than that of xylose because cellulose is more difficult to break down than hemicellulose [36]. Besides, negligible change of glucose yield (below 3.0%) was observed between 120 °C and 130 °C, although the reacted time was increased. The maximum yield of 5.7% was attained when pretreated at 150 °C for 15 min. This indicated that only a small amount of cellulose in the bagasse was degraded into glucose during the pretreatment, and it was also confirmed that the conditions used in our experiments only facilitated the hydrolysis of xylan, not cellulose. The overall trend of glucose yield was consistent with previous results [17,37].

Based on the above results, almost all of the xylan-type hemicelluloses were released from the cell wall of bagasse and further hydrolyzed into xylose and arabinose. The maximum sugar yield from the hydrolysis of bagasse hemicellulose, under the given conditions (120 °C, 10 min), was 93.2% and 90.7%, respectively, for xylose and arabinose. Severe reaction conditions (higher temperature or longer time) may have led to the excessive degradation of C5 sugars [36], which was also confirmed by the composition analysis of pretreated bagasse, as shown in composition analysis of the pretreated solid residual.

**Figure 3:**
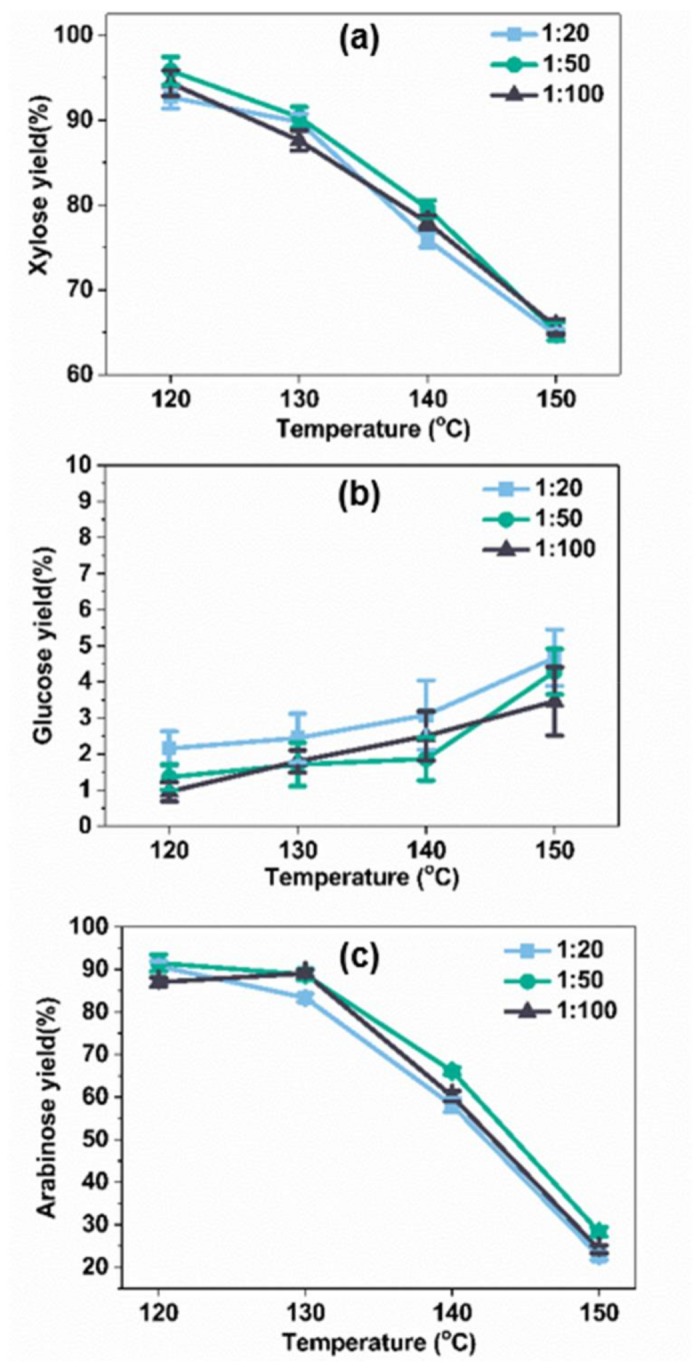
The effect of the solid–liquid ratio and temperature on sugar yields (oxalic acid 0.4 mol/L, 10 min, (**a**) xylose yield; (**b**) glucose yield; (**c**) arabinose yield).

Experiments were also performed to investigate the effects of the solid–liquid ratio on the yield of hydrolysate sugars, as displayed in Figure 3. Acid pretreatments with three different solid–liquid ratios (1:20, 1:50, and 1:100 g/mL) were carried out at different temperatures, with a constant reaction time of 10 min. For xylose and arabinose, their maximum yields of 95.7% and 91.5%, respectively, were achieved when pretreated at 120 °C, with a solid–liquid ratio of 1:50 g/mL. This could be because a higher solid–liquid ratio was beneficial to the formation of inhibitors [37,38]. Under these conditions (120 °C, solid–liquid ratio 1:50, 10 min, oxalic acid 0.4 mol/L), almost all of the xylan was hydrolyzed into xylose, without any noticeable side reactions. However, for glucose, the highest yield of 4.66% was obtained at 150 °C for 10 min, at a ratio of bagasse to water of 1:20. In comparison with Figure 2, it was found that the pretreatment temperature and time were the significant factors that contributed to the sugar yield.

### 2.3. Characterizations of the Untreated and Pretreated Bagasse

#### 2.3.1. Composition Analysis of the Pretreated Solid Residual

**Figure 4:**
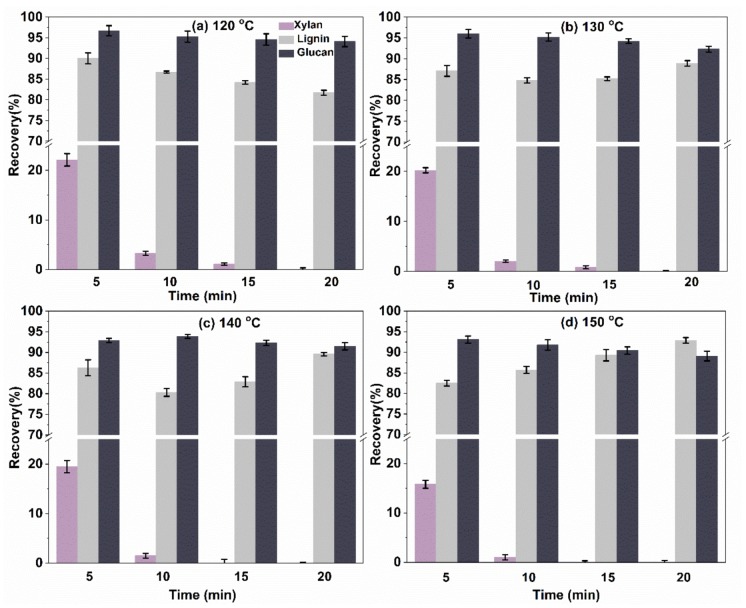
The effect of reaction temperature and time on the recovery of glucan, xylan, and lignin (1:20 solid–liquid ratio).

The major compositions of bagasse after pretreatment with microwave-assisted oxalic acid are shown in Figure 4. The xylan content of treated bagasse varied between 0.1% and 22.1%. With an increase in temperature and/or time, the xylan content of the residual solid was decreased significantly. This indicated that most of the xylan was released from the cell walls of bagasse, which was also proven by the differential thermogravimetry (DTG) curve (as shown in TGA and DTG analysis). It was reported that a xylan content of 0.07% in the residual solid was obtained from the wheat straw pretreatment (5% of oxalic acid, 210 °C, 20 min) [39]. However, in this work, a similar result could be achieved using a lower oxalic acid concentration (equivalent 3.6% oxalic acid) and under more mild conditions (120–150 °C, 15–20 min). This revealed that mild conditions facilitated the release and hydrolysis of xylan from the cell wall, leading to lower xylose content in the residual solid. On the contrary, harsh conditions would cause side reactions of xylose in the hydrolysate, as indicated in Figure 2. In view of the xylan mass balance, the xylose yield in the hydrolysate and xylan content left in the residual solid was 93.2% (120 °C, 10 min, a 1:20 solid–liquid ratio, Figure 2) and 3.3%, respectively, the corresponding xylan loss was only 3.5%, which confirmed that this pretreatment could realize the highly-selective dissolution and high-efficiency hydrolysis of xylan, in bagasse, to C5 sugars.

The glucan content of the residual solids was similar for all preset conditions, although there was a slight decrease in glucan content with the increase of temperature and time. This indicated that cellulose was not easily hydrolyzed by oxalic acid under the employed conditions, because of its crystal structure and hydrogen bonds [37,38].

Unlike xylan and glucan content, lignin content of the pretreated bagasse (from 80.3% to 92.9%) showed different trends, depending on the temperature. At 120 °C, lignin content decreased with increasing time, while at 130 °C and 140 °C, lignin content reached its minimum level within 10 min and then increased. At 150 °C, lignin content increased with increasing time. These phenomena could be attributed to the formation of water-insoluble humins, which are highly polymerized carbonaceous species generated by the reaction of furfural and xylose in the aqueous phase, or by the self-reaction of furfural in severe conditions [40].

#### 2.3.2. SEM Analysis

**Figure 5:**
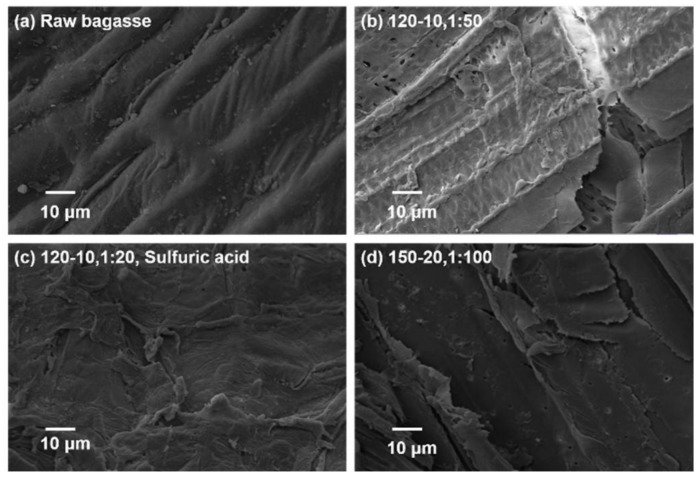
SEM of untreated and pretreated bagasse (120–10, 1:50 means temperature–time, solid–liquid ratio).

The surface morphology of raw (Figure 5a) and pretreated bagasse fibers (Figure 5b–d) is shown in Figure 5. The raw bagasse fibers had a smooth, more compact surface structure when compared to the pretreated fibers. During pretreatment, the fiber surface was disrupted to varying degrees. As can be seen in Figure 5b, the internal cell wall structure was exposed after microwave-assisted oxalic acid pretreatment at 120 °C for 10 min, but the fiber skeleton was still retained, since only xylan was removed. The fibers that were pretreated by sulfuric acid under the same temperature and time conditions as those of oxalic acid had more seriously damaged surfaces, due to the stronger acidity of sulfuric acid. Contrary to the moderate pretreatment (at 120 °C), the fiber surface was heavily damaged after pretreatment at 150 °C for 20 min, due to the solubilization of hemicellulose and partial removal of lignin from the cell wall [41]. Thus, more severe pretreatments led to greater destruction of lignocellulose. However, the corresponding xylose yield, as measured by HPLC, had only slightly increased or may have even decreased due to side reactions. This also confirmed that the pretreatment had a significant influence on sugar production.

#### 2.3.3. TGA and DTG Analysis

**Figure 6:**
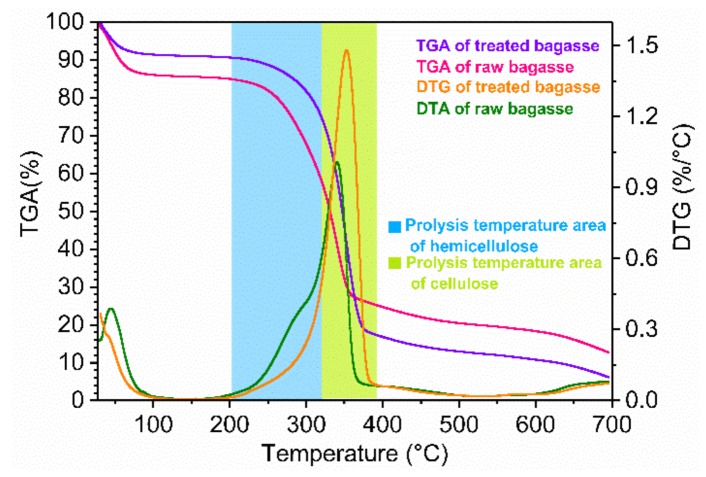
Thermal gravity analysis (TGA) and differential thermogravimetry (DTG) of the raw and pretreated bagasse fibers (120 °C, 10 min, 1:50, 0.4 mol/L oxalic acid).

TGA and DTG analyses were used to further evaluate the effects of the microwave-assisted acid pretreatment on bagasse fiber thermal degradation (as shown in Figure 6). There was a remarkable difference in thermal stability between the raw and pretreated bagasse fibers, indicating that the oxalic acid pretreatment had a crucial impact on the composition of the bagasse fibers. The main weight loss for raw and pretreated bagasse occurred at 250–360 °C and 280–375 °C, respectively. Raw bagasse contains a high content of lignin (24.5%) and xylan (23.4%). During pretreatment, almost all the hemicellulose and a certain amount of lignin were removed from the cell walls of the raw bagasse fibers, leading to higher thermal stability [42]. It was reported that the main pyrolysis temperature of hemicellulose ranged from 200 °C to 320 °C, while that of the cellulose was 320–400 °C [43,44]. The pyrolysis of the residual lignin mainly occurred at 200–500 °C.

The DTG curve of raw bagasse fibers had a shoulder at 280 °C and a peak at 340 °C. After microwave-assisted oxalic acid pretreatment, the shoulder disappeared, which revealed that most of the hemicellulose was degraded into small fractions [42]. Negligible peak variation showed that cellulose was only slightly affected by the acid pretreatment. At 700 °C, the weight of the solid residue was about 18% and 9% for raw and pretreated bagasse, respectively. This could be due to the lower lignin and ash content of the pretreated bagasse compared to the raw bagasse [45].

### 2.4. Enzymatic Hydrolysis Analysis

**Figure 7:**
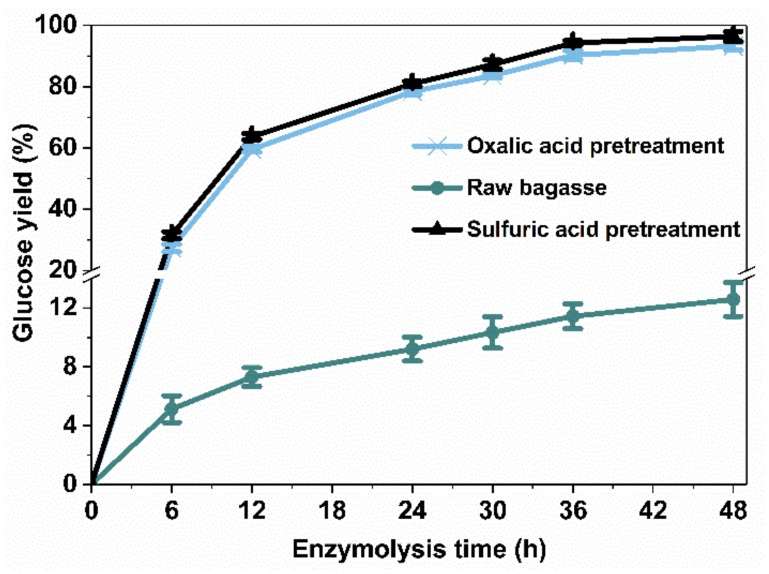
Time profiles of the enzymatic hydrolysis of raw bagasse and bagasse when pretreated with different acids.

Microwave-assisted acid pretreatments (120 °C, 10 min, 1:50 solid–liquid ratio, 0.4 mol/L oxalic acid and sulfuric acid) were first performed to remove the hemicellulose from bagasse in order to enhance enzymatic digestibility [46]. The glucose yield after the enzymatic hydrolysis of pretreated bagasse is shown in Figure 7. Both kinds of acid pretreatments played very important roles in promoting enzymatic hydrolysis. After 48 h of hydrolysis by cellulase (25 FPU/g dried pretreated bagasse), the maximum glucose yield of raw bagasse and pretreated bagasse by oxalic acid and sulfuric acid was 12.6%, 92.2%, and 95.0%, respectively. Previous analyses indicated that although most of the xylan-type hemicellulose was removed efficiently during the pretreatment process, within a short time, the remaining hemicellulose in the pretreated bagasse would hinder the interaction between cellulase and cellulose [47,48]. The pretreated bagasse exhibited looser, porous structures (as shown in Figure 5), which were favorable for increasing the accessibility of cellulose, and enhancing enzymatic hydrolysis efficiency. The glucose yield after sulfuric acid pretreatment was slightly higher than that of oxalic acid pretreatment, although the latter had a higher xylose yield. This can be explained by the fact that sulphuric acid has a stronger acidity than oxalic acid and causes more severe damage to the bagasse fiber structure (Figure 5c), which is favorable for the accessibility of cellulose to cellulase.

In the past decade, several pretreatment technologies have been used to improve enzymatic hydrolysis efficiency. Peracetic acid pretreatment has been used to accelerate the enzymatic hydrolysis of cellulose to glucose, with a conversion of over 80% being obtained [49]. Liu et al. evaluated the effects of FeCl_3_ pretreatment (160 °C for 20 min) on the enzymatic hydrolysis of corn stover. Their results showed that the conversion was up to 98%, with the enzyme dosage being as high as 60 FPU cellulase and 105.00 CBU β-glucosidase/g cellulose [50]. In another case, ethanol was used to pretreat bagasse at 195 °C for 30 min with 40% ethanol concentration, and a maximum glucose yield of 92.2% was achieved [51]. In this study, a high C5 sugar yield (xylose 95.7%, arabinose 91.5%) and a high glucose yield (92.2%) were both obtained via microwave-assisted oxalic acid pretreatment (120 °C, 10 min, 1:50 solid–liquid ratio) and enzymatic hydrolysis (25 FPU/g dried pretreated bagasse, 50 °C).

## 3. Materials and Methods

### 3.1. Materials

Sugarcane bagasse was provided by a sugar factory in Guangdong province, China. The main components of the extracted bagasse were (*w*/*w*): glucan 43.6%, xylan 23.4%, and total lignin 23.5% (acid insoluble 22.9% + acid soluble 0.6%). Sulfuric acid (98%, AR), hydrochloric acid (36.5%, AR) were purchased from the Tianjin Kermel Co., Ltd. (Tianjin, China). The sugar standards (d-xylose, d-glucose, l-arabinose etc.) were purchased from Sigma-Aldrich. Oxalic acid (≥99.5%, AR) and maleic acid (≥99.7%, AR) were obtained from the Shanghai LingFeng Chemical Agent Co., Ltd (Shanghai, China). Cellulase (Product No: C805042) was supplied by the Shanghai Macklin Biochemical Co., Ltd (Shanghai, China). All reagents were used without any purification.

### 3.2. Microwave-Assisted Acid Pretreatment of Sugarcane Bagasse

The sugarcane bagasse powder was suspended in acid solutions (oxalic acid, sulfuric, hydrochloric, or maleic acid) with a given solid–liquid ratio. Then the suspension was ultrasound treated (40 KHZ, 125 W) for 15 min at room temperature. Afterwards, microwave irradiation (600 W, GAS-800, Beijing Xianghu Science and Technology Development Reagent Co., Ltd., Beijing, China), was applied to heat the suspension to the target temperature with a heating rate of 10 °C/min. The heating-up time was not included in the reaction time. The microwave reactor automatically cooled for ten min after the reaction was complete. The solids were filtrated, washed with hot water, dried at 60 °C, and stored in a desiccant dryer for analysis of their thermal stability and surface properties. The liquids were filtered with a 0.22 μm nylon filter. All the experiments were performed in triplicate.

### 3.3. Enzymatic Hydrolysis of Pretreated Bagasse

The cellulose content of the pretreated bagasse was measured before the enzymatic hydrolysis. The hydrolysis was carried out in an air-bath shaker (50 °C and 200 rpm, Suzhou Pei Ying Experimental Equipment Co., Ltd, Jiangsu, China) for 48 h using the cellulase enzyme as a catalyst. 0.5 g of pretreated bagasse substrate was loaded with 50 mL of the 0.05 mol/L citrate buffer solution (pH 4.8), and cellulase was loaded at 25 FPU/g, based on the dried treated bagasse. One milliliter samples were taken at 6, 12, 24, 30, 36, and 48 h, inactivated with boiling water for 5 min, filtered with a 0.22 μm nylon filter, and then stored at −4 °C until analysis.

### 3.4. Analysis

The sugars in the liquid samples after both pretreatment and enzymatic hydrolysis were determined using HPLC, which was equipped with a Waters 1515 pump, an aminex column HPX-87H (BIO-RAD), and a Waters 2412 Refractive Index Detector. A solution of H_2_SO_4_ (5 mmol/L) was used as the mobile phase with a flow rate of 0.5 mL/min, and the column oven temperature was maintained at 60 °C.

The chemical compositions of the pretreated bagasse were determined according to the National Renewable Energy Laboratory (NREL) standard analytical method [52]. The component recovery ratios of the pretreatment and glucose yields after enzymatic hydrolysis were calculated as follows.

(1) $$\mathrm{Glucose}\;\mathrm{yield}=\frac{\mathrm{Glucose}\;\mathrm{in}\;\mathrm{enzymatic}\;\mathrm{hydrolysate}(g)\times 0.9}{\mathrm{Glucan}\;\mathrm{in}\;\mathrm{raw}\;\mathrm{material}(g)}\times 100\%$$

(2) $$\mathrm{Glucan}\;\mathrm{recovery}(\%)=\frac{\mathrm{Glucose}\;\mathrm{in}\;\mathrm{pretreated}\;\mathrm{residue}(g)\times 0.9}{\mathrm{Glucan}\;\mathrm{in}\;\mathrm{raw}\;\mathrm{material}(g)}\times 100\%$$

(3) $$\mathrm{Xylan}\;\mathrm{recovery}(\%)=\frac{\mathrm{Xylose}\;\mathrm{in}\;\mathrm{pretreated}\;\mathrm{residue}(g)\times 0.88}{\mathrm{Xylan}\;\mathrm{in}\;\mathrm{raw}\;\mathrm{material}(g)}\times 100\%$$

(4) $$\mathrm{Lignin}\;\mathrm{recovery}(\%)=\frac{\mathrm{Lignin}\;\mathrm{in}\;\mathrm{residue}(g)}{\mathrm{Lignin}\;\mathrm{in}\;\mathrm{raw}\;\mathrm{material}(g)}\times 100\%$$

The surface properties of pretreated bagasse were examined by a scanning electron microscope (SEM, S-4300, Hitachi, Tokyo, Japan) at 10 kV. The thermal stability of the raw material and solid residues were measured by thermogravimetric analysis (TGA, TA Q500, TA Instruments, New Castle, DE, USA), of which the maximum test temperature was 700 °C and the heating rate was 10 °C/min.

## 4. Conclusions

A feasible microwave-assisted oxalic acid pretreatment of bagasse was developed to efficiently produce xylose and arabinose sugars under mild conditions. The pretreatment described could substantially enhance the enzymatic hydrolysis efficiency. Among the four tested acids, oxalic acid had the best catalytic performance for the hydrolysis of xylan-type hemicellulose into C5 sugars, followed by sulfuric acid, hydrochloric acid, and maleic acid in the given conditions. The results showed that acid concentration, temperature, and time had significant effects on C5 sugar yield during pretreatment. The highest xylose yield of 95.7% and arabinose yield of 91.5% could be achieved at 120 °C for 10 min with a solid–liquid ratio of 1:50 using 0.4 mol/L oxalic acid. The enzymatic hydrolysis efficiency was greatly improved, and the corresponding glucose yield was 92.2% (oxalic acid pretreatment).
